# Comparative evaluation of antioxidant activity, total phenolic content, anti-inflammatory, and antibacterial potential of *Euphorbia*-derived functional products

**DOI:** 10.3389/fphar.2024.1345340

**Published:** 2024-02-22

**Authors:** Rania Benjamaa, Hamza Elbouny, Hajare Errati, Abdelkarim Moujanni, Neha Kaushik, Ravi Gupta, OumKeltoum Ennibi, Boubker Nasser, Eun Ha Choi, Nagendra Kumar Kaushik, Abdelkhalid Essamadi

**Affiliations:** ^1^ Laboratory of Biochemistry, Neurosciences, Natural Resources and Environment, Faculty of Sciences and Technologies, Hassan First University of Settat, Settat, Morocco; ^2^ Laboratory of Biochemistry, Department of Biology, Faculty of Sciences and Technology, University Moulay Ismail, Meknes, Morocco; ^3^ Laboratory of Agri-Food and Health, Faculty of Sciences and Techniques, Hassan First University, Settat, Morocco; ^4^ Department of Biotechnology, College of Engineering, The University of Suwon, Hwaseong, Republic of Korea; ^5^ College of General Education, Kookmin University, Seoul, Republic of Korea; ^6^ Department of Periodontology, Faculty of Medicine Dentistry, Research Laboratory on Oral Biology and Biotechnology, Mohammed V University in Rabat, Rabat, Morocco; ^7^ Department of Electrical and Biological Physics, Plasma Bioscience Research Center, Kwangwoon University, Seoul, Republic of Korea

**Keywords:** *Euphorbia resinifera O. Berg: Euphorbia officinarum subsp. echinus (Hook.f. and Coss.) Vindt*, functional food products, antioxidant effect, phenolic compounds, antiinflammatory potential, antibacterial effect

## Abstract

This study assessed the medicinal properties of *Euphorbia resinifera O. Berg (E. resinifera)* and *Euphorbia officinarum subsp echinus (Hook.f. and Coss.) Vindt* (*Euphorbia echinus*, known for their pharmaceutical benefits. Extracts from their flowers, stems, propolis, and honey were examined for phenolic content, antioxidant, anti-inflammatory, and antibacterial activities. Total phenolic content (TPC), total flavonoid content (TFC), and total condensed tannin (TCC) were determined using specific methods. Antioxidant potential was assessed through various tests including DPPH, FRAP, ABTS, and Total antioxidant capacity. Anti-inflammatory effects were evaluated using phenol-induced ear edema in rats, while antibacterial activity was measured against Gram-positive (*Staphylococcus aureus* ATCC 6538) and Gram-negative (*E. coli* ATCC 10536) bacteria. Among the extracts, the aqueous propolis extract of *E. resinifera* demonstrated exceptional antioxidant capabilities, with low IC_50_ values for DPPH (0.07 ± 0.00 mg/mL) and ABTS (0.13 ± 0.00 mg/mL), as well as high TAC (176.72 ± 0.18 mg AA/mg extract) and FRAP (86.45 ± 1.45 mg AA/mg extract) values. Furthermore, the anti-inflammatory effect of *E. resinifera* propolis extracts surpassed that of indomethacin, yielding edema percentages of 3.92% and 11.33% for the aqueous and ethanolic extracts, respectively. Microbiological results indicated that the aqueous extract of *E. resinifera* flower exhibited the most potent inhibitory action against *S. aureus*, with an inhibition zone diameter (IZD) of 21.0 ± 0.00 mm and a minimum inhibitory concentration (MIC) of 3.125 mg/mL. Additionally, only *E. resinifera* honey displayed the ability to inhibit *E. coli* growth, with an inhibition zone diameter of 09.30 ± 0.03 mm and a MIC of 0.0433 mg/mL.

## 1 Introduction

The exploration of natural products, specifically medicinal plants, offers a promising pathway for discovering innovative pharmacophores in the endeavor to design new drugs for alleviating human suffering (Mahomoodally et al., 2018). Plants function as natural reservoirs of bioactive compounds, generating a diverse range of phytochemicals that harbor potential therapeutic properties (Koehn and Carter, 2005; Shen, 2015). The essential task of harnessing and probing into these compounds is critical for revealing their medicinal capacities. Importantly, natural products have demonstrated significant success in the realm of drug development, with more than 100 novel products currently undergoing clinical development, notably as anti-cancer agents and anti-infectives (Harvey, 2008).

Oxidative stress and inflammation are two major outcomes of stress conditions in animals, including humans. While reactive oxygen species (ROS) generation and cellular antioxidant capabilities are out of equilibrium, causing oxidative stress (Finaud et al., 2006), *inflammation is the result of the reaction of the immune system to* internal or external injury and infection (DeMaio et al., 2022). It is now clear that a variety of chronic diseases, including diabetes, hypertension, alcoholic liver disease, cancer, chronic kidney disease, aging, obesity, and atherosclerosis, are linked to oxidative stress and inflammation (Biswas and Lopes de Faria, 2006; Biswas et al., 2007; Federico et al., 2007; Cachofeiro et al., 2008; Krishnan, 2010; Ambade and Mandrekar, 2012; Bondia-Pons et al., 2012). During an inflammatory reaction, activated macrophages and neutrophils trigger the release of ROS at the site of inflammation (Fialkow et al., 2007) On the other hand, oxidative stress is one of the triggers of the inflammatory process; an excessive release of free radicals can release an inflammatory response in an attempt to eliminate them. The exploration for compounds that target both inflammation and oxidative stress concurrently is viewed as a crucial approach in mitigating or managing ailments linked to prolonged inflammatory states. This is because solely addressing one aspect may not always yield optimal results in controlling or treating such conditions (Biswas, 2016).

*Euphorbia* is among the most extensive genera within the Euphorbiaceae family (Barla et al., 2006) and is represented by 2,000 species (Jassbi, 2006). Members of this family are found throughout the world, especially in Southern, Eastern, and Northeastern Africa, North and Central Mexico, and Western Asia (Webster, 1994). The genus is characterized by a large number of bioactive compounds (Pascal et al., 2017). *Euphorbia resinifera O. Berg* (*E. resinifera*) and *Euphorbia officinarum subsp. echinus. (Hook.f. and Coss.) Vindt* (*Euphorbia echinus*), locally known respectively under the names of *Zeggoum* (Moujanni et al., 2018) and *Deghmous* (Chamkhi et al., 2022)*,* are two species that are endemic to Morocco and North Africa, respectively (Moujanni et al., 2017). In the past, these species have been extensively used in the customary Moroccan clinical system (Bourhia et al., 2019; Alami et al., 2020) to treat ophthalmological diseases, intestinal parasites, skin, cancer, cyst (Chamkhi et al., 2022), snake bite poisoning and rheumatism (Benmehdi et al., 2013)**.** Therefore, efforts have been made in the past to understand the medicinal properties of these plants. However, the majority of those studies were focused on the latex component only, while the information on the other components is scanty (Fattorusso et al., 2002; Avila et al., 2010; Smaili et al., 2017; Badaoui et al., 2022).

In this context, our research aims to provide a comprehensive analysis of the phenolic profile of these plants and to evaluate the efficacy of their extracts as potent antioxidant, anti-inflammatory, and antibacterial agents. By extending the analysis beyond the traditionally studied latex component, our study seeks to contribute valuable insights into the medicinal properties of *E. resinifera* and *E. echinus*, thus filling an existing knowledge gap in the field of natural product chemistry and pharmacology.

## 2 Materials and methods

### 2.1 Collection of plant

The aerial parts of *E. resinifera* and *E. echinus,* such as stems and flower petals, were collected from the fields of Ouaouizeght (harvest location according to Merchich northern Morocco X: 410804 Y: 179306) in July and Tiznit (harvest location according to Merchich Sahara nord X: 80000 Y: 274000) in August, respectively, in Morocco during their flowering season in 2020 under the control of the High Commission for Water and Forests in two regions. The wild plants were identified at the Scientific Institute - Department of Botany in Rabat, Morocco; the voucher reference codes for *E. resinifera* is RAB114129, and for *E. officinarum subsp*. *echinus* is RAB114130.

The collection process adhered to stringent environmental recording practices. Specifically, the temperature during the collection in Ouaouizeght was recorded at 42°C, and in Tiznit, it was recorded at 50°C.

The honey and propolis were collected directly from the hives of the bee farms to avoid contamination of samples. After collection, samples were kept ambient.

### 2.2 Extract preparation

The leaves stems, and propolis were left to dry at ambient temperature in the dark and powdered. For ethanol extraction, 2 g of all samples was extracted with 96% ethanol (20 mL) (Merck-Germany; CAS Number: 64-17-5) and distilled water (40 mL) by maceration for 24 h to obtain aqueous and ethanol extracts. After filtration, the ethyl alcohol was completely evporated using rotary evaporator at 45°C temperature to get dry extract, and for aqueous extract, sample was lyophilized in a freeze dryer at 10 m Torr pressure at −50°C. The residues were dissolved in dimethylsulphoxide (DMSO, Merck-Germany; CAS Number: 67-68-5) and then kept at freezer for further experiments.

### 2.3 Analysis of plant extract yield

The determination of extraction yield for leaves, stems, and propolis followed a standardized formula:

$$Y\;\left(\%\right)=\left(\frac{\text{ME}}{\text{MP}}\right)x\;100$$

Whereas, Y (%) is percent yield, MP is plant mass or propolis compund (g) and ME is extract mass (g).

### 2.4 Analysis of total phenolic content

#### 2.4.1 Evaluation of total phenolic content

The total phenolic content (TPC) was quantified using the Folin-Ciocalteu reagent (Sigma-Aldrich, Cat. No. F9252), a method reported previously (Laličić-Petronijević et al., 2016). In brief, 250 µL of propolis extract 1 mg/1 mL DMSO (Merck-Germany; CAS Number: 67-68-5) and honey extract (1 g/1 mL distilled water) were amalgamated with 250 µL of Folin-Ciocalteu (Sigma-Aldrich, St Louis, United States) (diluted in 1:10 with water for 3 min) and then 2 mL 7.5% sodium carbonate solution (Merck-Germany; CAS Number: 497-19-8) was added to the mixture and thoroughly mixed. Following incubation for 30 min, the absorbance of sample and blank solvent was detected at 765 nm wavelenth using a UV-Vis spectrophotometer. The concentration of TPCs was showed as mg gallic acid (Sigma-Aldrich, St. Louis, MO, United States, Cas Number: 149-91-7) equivalent (GAE) per Gram of extract (mg GAE/g extract) for plant extracts and as mg GAE/100 g for propolis and honey. The equation for the standard curve of gallic acid was Y = 0.0061X+ 0.00418, and the coefficient of correlation was *R* ^2^ = 0.9967.

#### 2.4.2 Evaluation of total flavonoid content

The colorimetric method based on aluminum chloride was used for the quantification of total flavonoid contents (TFC), as described previously (Chang et al., 2002)**.** The plant and propolis extract were made at the concentration of 1 mg/mL (DMSO, Merck-Germany; CAS Number: 67-68-5), and the honey solution was made at the concentration of (1 g/mL distilled water). Briefly, 0.5 mL of each sample was added to 1.5 mL of 95% ethanol (Merck-Germany; CAS Number: 64-17-5), 0.1 mL of 10% aluminum chloride (Sigma-Aldrich Chemical Co. St Louis, MO, United States, CAS Number: 7446-70-0), 0.1 mL of 1 M potassium acetate (Merck-Germany; CAS Number: 127-08-2), and 2.8 mL of water and incubated at ambient temperature in darkness about 30 min. The optical density was mesured at 415 nm wavelenght using a UV-Vis spectrophotometer against the blank. Flavanoid compunds was showed as mg Quercetin (Merck-Germany; CAS Number: 6151-25-3) equivalent (QE) per Gram of extract (mg QE/g extract) for plant and mg GAE/100 g for propolis and honey extracts from a standard curve using equation Y = 0.0054X+0.0392 with *R* ^2^ = 0.9959. The measurements were conducted three times for each data point.

#### 2.4.3 Determination of condensed tannin content

The concentration of condensed tannins (TCC) or proanthocyanidins was analyzed using the vanillin/HCl technique, as previously detailed (Rebaya et al., 2015). To 400 µL of the diluted sample, 3 mL of methanol vanillin solution (4%, w/v) (Merck-Germany; CAS Number: 121-34-6) and 1.5 mL of concentrated hydrochloric acid (Merck-Germany; CAS Number: 7647-01-0) were mixed to each sample. Following a 15-min incubation at ambient temperature, the absorbance at 500 nm was recorded. The concentration of condensed tannins was indicated in mg of catechin (Merck-Germany; CAS Number: 18829-70-4) equivalent per g or per 100 g of the extract with an equation of Y = 0.0052X+ 0.0056 with *R* ^2^ = 0.9984. All measurements were conducted in triplicate.

### 2.5 *In vitro* antioxidant screening

#### 2.5.1 Analysis of DPPH scavenging activity

DPPH (2,2-diphenyl-1-picrylhydrazyl), radical scavenging activity was analysed using the method of Blois, M. S. (1958) (Blois, 1958). Briefly, 2 mL of DPPH (Sigma-Aldrich Co., St. Louis, MO, United States, Cas Num: 84077-81-6) methanolic solution (0.1 mM) (Merck-Germany; CAS Number: 67-56-1) was added to 0.1 mL of the sample extract at different concentrations or a control. After 30 min in darkness and at ambient temperature, the absorbance of the reaction mixture was measured at wavelength of 517 nm. For the blank, the extract was substituted with methanol, and ascorbic acid (Sigma-Aldrich, Cas Number: 50-81-7) as a positive control was used. The extract’s capacity to scavenge DPPH free radicals was determined by employing the formula for calculating the concentration of the sample that results in 50% inhibition:

$$\text{Scavenging activity }\left(\%\right)=\left(\frac{\left(\text{OD}\;\text{control}\;–\;\text{OD}\;\text{sample}\right)\;}{\text{OD}\;\text{contro}}\right)x\;100$$

#### 2.5.2 ABTS analysis

The capability of ABTS, 2,2′-azino-bis (3-ethylbenzothiazoline-6-sulfonic acid) to scavenge radicals was assessed following the methodology outlined as described earlier (Re et al., 1999). Briefly, an aqueous solution of potassium persulfate (2.45 mM) and a 7 mM aqueous ABTS solution were mixed to prepare the assay reagent. This mixture was left in the dark overnight. Prior to use, the assay reagent was diluted with EtOH to achieve 0.70 (±0.01) absrobance at 734 nm wavelength. Then, 100 µL of extract sample at different concentrations were blended with 2 mL of the ABTS solution (7 mM). Afterwords samples was incubated for 10 min, and the absorbance was detected at 734 nm wavelength. Ethanol (Merck-Germany; CAS Number: 64-17-5) served as the control, and a range of ascorbic acid solutions (Merck-Germany; CAS Number: 50-81-7) were employed for calibration. The percentage of inhibition was computed utilizing the formula outlined in the DPPH assay, and the outcomes were expressed as IC_50_ values.

$$\left(\%\right)\text{ inhibition}=\left(\frac{\left(\text{OD}\;\text{control}\;–\;\text{OD}\;\text{sample}\right)\;}{\text{OD}\;\text{contro}}\right)x\;100$$

#### 2.5.3 Phosphomolybdenum assay

Total antioxidant capacity (TAC) was assesed via phosphomolybdenum method as described earlier (Prieto et al., 1999)**.** An aliquot (0.3 mL) of a 1 mg/mL sample solution was added in 2.7 mL of phosphomolybdenum reagent, which consists sodium phosphate (28 mM) (Merck-Germany; CAS Number: 10049-21-5) and ammonium molybdate (4 mM) (Merck-Germany; CAS Number: 13106-76-8) in sulphuric acid (0.6 M) (Merck-Germany; CAS Number: 7664-93-9) in a 4 mL vial, and incubated for 90 min at 95°C. The optical density of the mixture was read spectrophotometrically at 695 nm wavelength against the blank. The obtained results were represented as mg ascorbic acid equivalents/g extract utilizing a standard calibration curve of ascorbic acid (Merck-Germany; CAS Number: 50-81-7), using equation Y = 0.0047X- 0.0259 with *R* ^2^ = 0.9975. All experiments were carried out in triplicates.

#### 2.5.4 Ferric reducing antioxidant power assay (FRAP)

The FRAP analysis, following the protocol outlined by Benzie and Strain (1999), was employed in this study (Benzie and Strain, 1999). Briefly, acetate buffer (0.3 M at pH = 3.6), tripyridyltriazine (TPTZ) solution (10 mM) produced in HCl (40 mM) (Merck-Germany; CAS Number: 7647-01-0), and ferric chloride solution (20 mM) (Merck-Germany; CAS Number: 7705-08-0) were mixed to make the assay working solution. A 50 µL test sample (mg/mL) for plant and propolis extract both, and (1 g/mL) for honey, was reacted with 2 mL of FRAP reagent and incubated for 10 min at ambient conditions. The optical density of the mixtures was detected at 593 nm wavelength. In this assay, ascorbic acid (Merck-Germany; CAS Number: 50-81-7) used to make calibration curve or standard to facilitate result comparison with other methods, using equation Y = 0.0149X-0.0073, where *R* ^2^ = 0.9999. The results of this assay, which was performed in triplicate, were given as mg of ascorbic acid equivalents per g of dried samples for the plant and propolis and mg of ascorbic acid equivalents per 100 g for the honey.

### 2.6 Anti-inflammatory assay in rat models

#### 2.6.1 Animals

In this study, male Wistar rats was used weighing between 100 and 115 g. The animals were kept in a controlled conditions with a 12 h light/dark cycle, a temperature of 24°C ± 4°C was maintained, with unlimited access to food and drink. Prior to the experiment, rats spent about a week to adopt the laboratory conditions. The pharmaceutical research committee, FSTE, Moulay ISMAIl University’s criteria for the use of laboratory animals (ARECFSTE-12/2020) were followed for conducting animal experiments.

#### 2.6.2 Phenol- induced rat ear edema

The anti-inflammatory assay was conducted in accordance with the previously outlined method (Rodrigues et al., 2016). The animals, weighing between 100 and 120 g, were sorted into six uniform groups. Phenol, indomethacin, as well as extracts from stems, flowers, propolis, and honey were mixed in acetone and administered to both the inner and outer surfaces of the ear. Each group (n = 6) received 10 µL of phenol (15%) (Merck-Germany; CAS Number: 108-95-2) on the right ear, while the left ear was treated with acetone (Merck-Germany; CAS Number: 67-64-1) only (Vehicle). Subsequently, following the application of the irritant, indomethacin (1 mg/mL) was administered on the right ears, along with honey (1 mg/mL) and various extracts (1 mg/mL). An hour later, the ear thickness was gauged using a digital caliper. The edema percentage was determined using the subsequent formula:

$$\%\text{ of edema}=\frac{\left(T1-T0\right)}{T1}X\;100$$

Where T0 represents the ear thickness prior to phenol application and T1 represents the ear thickness an hour later.

### 2.7 Antibacterial activity

#### 2.7.1 Microorganisms

The Microbiology Laboratory of the Pharmaceutical and Veterinary Products Division of the National Office of Food Safety (ONSSA), Rabat, Morocco, gifted the Gram-positive (*Staphylococcus aureus*; ATCC 6538) and Gram-negative (*E. coli*; ATCC 10536) bacteria, which were used to perform the antibacterial properties of various *Euphorbia* extracts.

#### 2.7.2 Antibiotic susceptibility test

The disk diffusion method was used to evaluate the antibiotic susceptibility of pathogens, using Muller–Hinton agar as per the standards reported by the Clinical and Laboratory Standards Institute (CLSI). The filter paper discs (antibiotics) were placed on the top of agar that had been inoculated with test bacteria. After 20 h, the inhibition zone diameter was calculated and compared with that of the samples. The antibiotic disks used were: Ampicillin (10 µg) (AMC), Céfotaxim (30 µg) (CTX), Ciprofloxacin (5 μg) (CIP), Fosfomycin (50 µg) (FOS), Co-trimoxazole (25 µg) (SXT), Penicillin (6 µg) (PEN), Nalidixic acid (30 µg) (NAL), Spectinomycin (100 µg) (SPT), Doxycycline (30 µg) (DO), Kanamycin (30 µg) (KMN), Nitrofurantoin (300 µg) (FTN), Enrofloxacin (5 µg) (ENR), Bacitracin (6 µg) (B), Tétracyclin (30 µg) (TET), Streptomycin (10 µg) (SMN), and Cefepim (30 µg) (CEF).

#### 2.7.3 Well diffusion method

The antimicrobial potential against bacterial strains were tested according to the described method (Das et al., 2013). In brief, 100 µL of bacterial strain corresponding to 0.5 of the McFarland standard (1.5 × 10^8^ CFU/mL) was spread with a non-toxic swab on Petri dishes containing MH (Mueller–Hinton) medium (Sigma-Aldrich, Chemie GmbH, Darmstadt, Germany). Three wells (with a diameter of 6 mm) were punched off by a cork-borer, and each well was then filled with 100 µL of 100 mg/mL of plant and propolis extract and (100, 50, 25% and 6.25% (v/v)) honey. The plates were kept for growth in incubator for 24 h at 37°C. The antibacterial screening was evaluated by inhibition zone diameter (IZD) measured in mm. The negative control was the solvent 5% (DMSO, Merck-Germany; CAS: 67-68-5), for the ethanolic extracts and distilled water for the aqueous extracts (Atef et al., 2019).

#### 2.7.4 Determination of minimum inhibitory concentration

The minimum inhibitory concentration (MIC) values were calculated by a broth dilution method. Briefly, 100 µL of Mueller Hinton broth (BMH) (Sigma-Aldrich, Chemie GmbH, Darmstadt, Germany) was added to each well of a multi-well plate, followed by the mixing of 100 µL of stock solution of each sample in the first column. A series of dilutions of reason 1/2 of the stock solution was carried out until the wells of Column 10 in order to have a range of concentrations varying from 50 mg/mL to 0.0976 mg/mL for plant extracts and from 5.55 mg/mL to 0.0108 mg/mL. The bacterial culture was adjusted to an absorbance equivalent to 0.5 McFarland, and 10 µL was introduced into each well of the plate except those of column 12. Column 11 was a positive growth control (tested strain and BMH), and column 12 corresponded to negative control (BMH without inoculum). Each assay was performed in triplicates. After incubation, 20 µL of an aqueous 2, 3, 5-triphenyl tetrazolium chloride (MTT) dye (Merck-Germany; CAS Number: 298-96-4) was added to the microplate wells and kept for 4 h in incubator. The MIC of the extract was established based on the lowest concentration that exhibited no microorganism growth, as indicated by the transition in color from yellow to pink (Oliveira et al., 2007).

#### 2.7.5 Minimum bactericidal concentration

To establish the minimum bactericidal concentration (MBC), a loopful from wells exhibiting no observable bacterial growth was incubated on Mueller-Hinton agar (Sigma-Aldrich, Chemie GmbH, Darmstadt, Germany) for 24 h at 37°C. The MBC value was expressed as the minimum dose of the antimicrobial agent that eradicates over 99.9% of the bacteria (Balouiri et al., 2016). The MBC/MIC ratio was determined to evaluate if the extracts under study had bacteriostatic or bactericidal effects. When the ratio was larger than four, the effect was termed bacteriostatic; when it was less than or equal to four, it was considered bactericidal (Djihane et al., 2017).

### 2.8 Statistics

Here, we employed a three-factor analysis of variance (ANOVA) to investigate the interplay among three key variables: *Euphorbia* species, *Euphorbia* products, and extract types, and their collective impact on the yield of extraction, phenolic compound concentration, and antioxidant activity. Our decision to use this statistical approach allowed us to ascertain whether these factors interact significantly to influence the observed outcomes (as shown in Table 1). Notably, we focused on the main effects of each factor, such as the different *Euphorbia* species (*E. resinifera* and *E. echinus*), various *Euphorbia* products (flowers, stems, and propolis), and different extraction types (water and ethanol). The analysis of variance demonstrated varying degrees of significance for the main effects of each factor on the response variables, including yields, TPC, TFC, TCC, TAC, FRAP, DPPH and ABTS radical scavenging activity. Additionally, the analysis identified certain significant interactions between factors, such as species-product (SpP), species-extract (SpExt), product-extract (PExt), and species-product-extract (SpP*Ext), which further elucidated how combinations of these factors contributed to specific effects on the investigated parameters. The results obtained through this comprehensive analysis shed light on the complex relationships between *Euphorbia* species, products, and extraction types and their collective influence on the biochemical properties of the extracts.

**TABLE 1:** Results of three-factorial analysis of variance ANOVA for yield percentage, phenolic content, and antioxidant potential of different *Euphorbia* extracts.

Factors	Factor level	Yield %	TPC	TFC	TCC	TAC	DPPH	ABTS	FRAP
Species (SP)	*E. resinifera*	20.00 a	62.00 a	27.3 a	1.78 a	84.65 a	1.06 a	1.41 az	41.35 a
	*E. echinus*	14.55 a	21.31 b	8.85 b	0.42 b	49.90 b	1.71 b	3.30 b	9.35 b
*Euphorbia* Product (P)	flowers	17.19 a	24.19 a	43.73 a	1.22 a	60.35 a	0.49a	3.31 b	27.32 ab
	stems	9.29 ab	8.56 a	10.55 ab	0.25a	45.29 a	3.19b	2.86 b	4.65 a
	propolis	25.35 b	21.57 a	70.69 b	1.84 a	96.20 b	0.49 a	0.89 a	44.07 b
Extract (Ext)	Water	12.54 a	41.50 a	12.21 a	1.52 a	62.98 a	1.83 a	2.10 a	21.73 a
	Ethanol	22.01b	41.82 a	24.01 b	0.68 a	71.59 a	0.95 a	2.60 a	28.97 a
Significance	Sp	0.227ns	0.002*	<0.001*	0.043*	0.006*	0.237ns	0.003*	<0.001*
	P	0.009*	0.047*	0.001*	0.152ns	0.003*	<0.001*	0.004*	0.001*
	Ext	0.032*	0.982 ns	0.034*	0.217 ns	0.520 ns	0.105 ns	0.465 ns	0.445 ns
	Sp*p	0.943 ns	<0.001*	<0.001*	0.023*	0.002*	0.859 ns	0.027*	<0.001*
	Sp*Ext p*Ext	0.123 ns	0.813 ns	0.730 ns	0.057 ns	0.561 ns	0.198 ns	0.004*	0.810 ns
	Sp*P* Ext	<0.001*	0.160 ns	0.117 ns	0.003*	0.005*	0.001*	0.372 ns	0.217 ns
		<0.001*	<0.001*	<0.001*	<0.001*	<0.001*	<0.001*	<0.001*	<0.001*

Data was evaluated via three-way ANOVA, factors: species, euphorbia products and extracts, followed by Tukey HSD, test (mean, n = 3). Identical letters indicate that values do not differ significantly. Asterisks (*) indicate significantly influential factors.

*E. Euphorbia resinifera O. berg; E. echinus: Euphorbia officinarum subsp. Echinus (Hook.f. and Coss.) vindt* ***;*** Ns, not significant; SP, species; Ext, Extract; *p*, product; TPC, total phenolic content; TFC, total flavonoid content; TCC, condensed tannin contents. TAC, total antioxidant capacity; DPPH, (2,2-diphenyl-1-picrylhydrazyl), radical scavenging activity; ABTS, 2,2′-azino-bis (3-ethylbenzothiazoline-6-sulfonic acid); FRAP, ferric reducing antioxidant power.

## 3 Results

### 3.1 Yield of extraction

**FIGURE 1:**
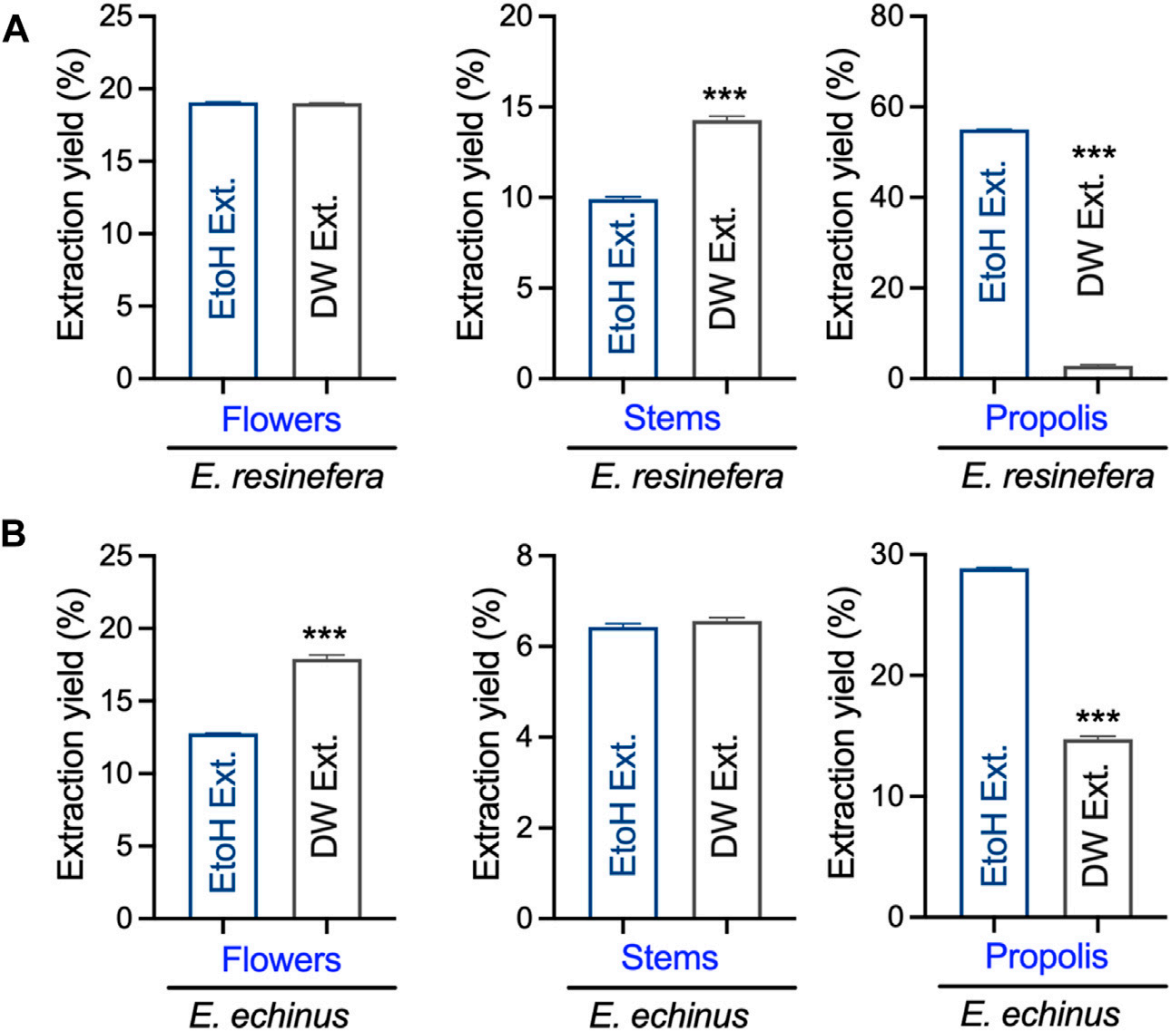
The yield obtained from the extraction of stems, flowers, and propolis of *E. resinifera* and *E. echinus*. EtoH Ext represents ethanolic extracts, while DW Ext indicates the water extracts prepared from different *Euphorbia* species. ***p < 0.001. **(A)** *E. resinifera* extract and **(B)** *E. echinus* extract.

Initially, we assessed the overall yield of each extract derived from the flower stems and propolis of both *E. resinifera* and *E. echinus* through maceration using ethanol and water (see Figure 1). Notably, the ethanol extract from *E. resinifera* propolis demonstrated the highest extraction yield (55.00% ± 0.03), surpassing all other extracts, including its aqueous counterpart (2.80% ± 0.19) (*p* ˂0.001), which exhibited the lowest yields among all extracts. In the case of *E. echinus*, the aqueous extracts from flowers exhibited a superior extraction yield (17.90% ± 0.61) compared to the ethanolic extract (12.79% ± 0.01) (*p* ˂0.001). Interestingly, there was no discernible change in the extraction yield of stems from both aqueous and ethanolic extracts. Concerning propolis, the extraction yields in both species were notably higher in ethanol (28.87% ± 0.03) than in water (14.73% ± 0.15) (*p* ˂0.001) (see Figure 1 and Supplementary Table S1). These findings underscore the critical influence of solvent choice on extraction efficiency, providing practical insights for future protocols and emphasizing the need for tailored approaches in extracting bioactive compounds.

### 3.2 Total phenolic, flavonoid, and condensed tannin contents

**FIGURE 2:**
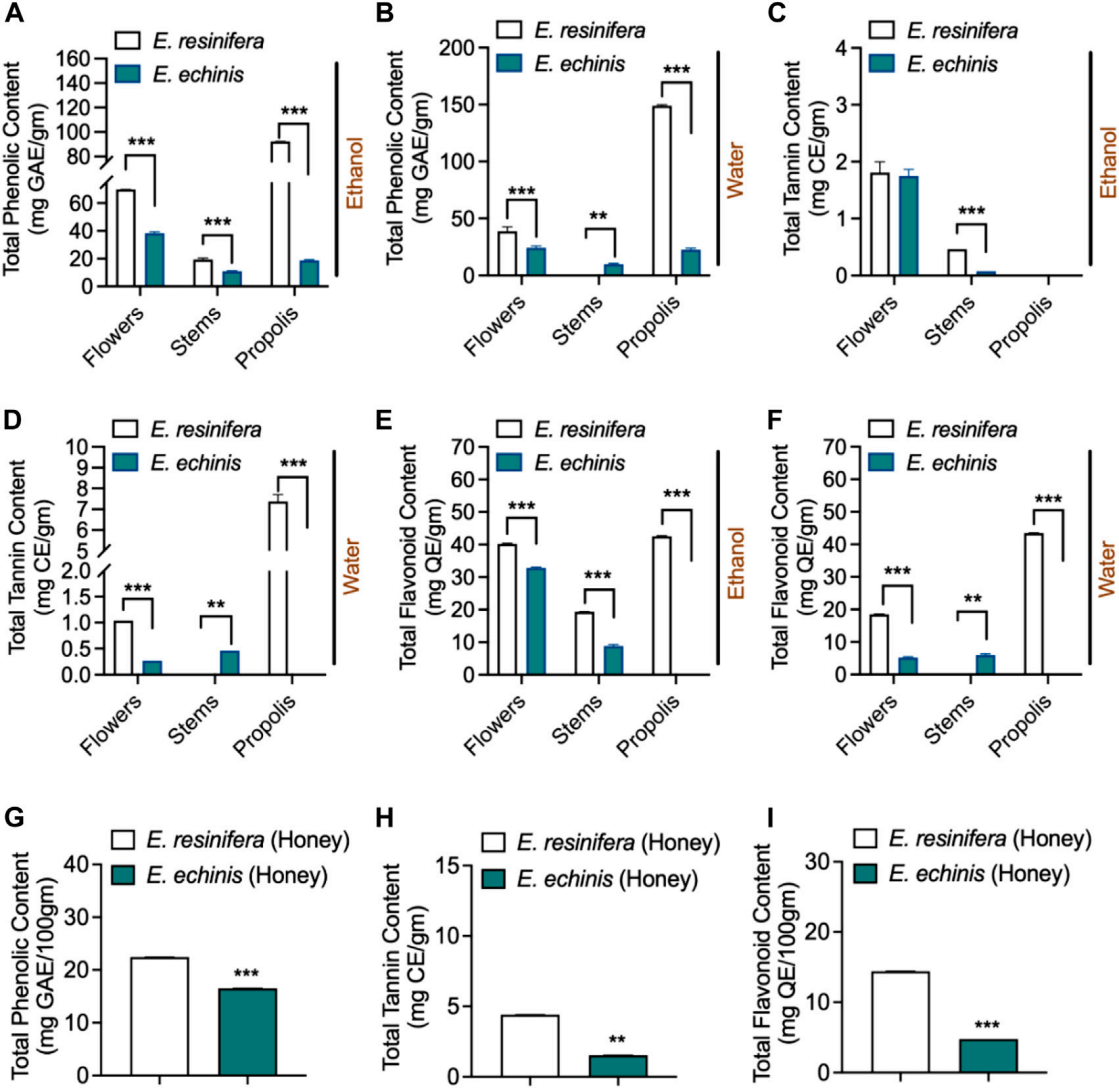
Phychemical characterization. The content of phenolic compounds: **(A)** ethanolic extracts. **(B)** aqueous extracts. **(C)** ethanolic extracts. **(D)** aqueous extracts. **(E)** ethanolic extracts. **(F)** aqueous extracts **(G)** Honey. The content of flavonoids. **(H)** Honey: in two *Euphorbia* species. **(I)** Honey. The content of condensed tannins. One-way ANOVA was employed to calculate the data independently for each species and factor (product and extract), after which the Tukey HSD test (mean, n = 3) was performed. **p ˂0.01 and ***p < 0.001.

Phenolic compounds, recognized as significant secondary metabolites in diverse plant organisms (Santos-Buelga et al., 2012), were investigated in ethanolic and aqueous extracts of flowers, stems, and propolis of two *Euphorbia* species, as well as honey (Figure 2, Supplementary Tables S2, S3). The ethanolic extracts demonstrated elevated total phenolic (TPC) levels in flowers, stems, and propolis compared to their aqueous counterparts. *Euphorbia resinifera* displayed markedly elevated levels of total phenolic (TPC) in flowers, stems, and propolis when extracted using either aqueous or ethanolic methods, exhibiting notable significance (*p* ˂0.001). However, an exception was observed in the aqueous extract of *E. resinifera s*tems, which yielded the lowest extraction levels, and this difference was statistically significant (*p* ˂0.01).

It is noteworthy that the aqueous extract of *E. resinifera* propolis exhibited the highest TPC content at 148.12 ± 0.14 mg GAE/g, while the aqueous extract of *E. resinifera* stems displayed the lowest TPC content at 0.72 ± 0.18 mg GAE/g. Substantial variations in TPC were observed within the same species across different plant parts and between the two *Euphorbia* species (Figures 2A, B). In terms of condensed tannin (CT), the aqueous extract of *E. resinifera* propolis exhibited the highest concentration (7.38–0.19 mg EC/g), followed by stems. However, ethanolic extracts of flowers for both species did not show significant differences, with values of 1.81 ± 0.11 mg CE/g and 1.75 ± 0.68 mg CE/g for *E. resinifera* and *E. echinus*, respectively. Notably, condensed tannins were absent in *E. echinus* propolis (Figures 2C, D and Supplementary Table S2).

Total flavonoid content (TFC) revealed higher extraction yields in ethanolic extracts compared to aqueous extracts. *Euphorbia resinifera* propolis exhibited the highest TFC content (42.55 ± 0.11 to 43.42 ± 0.06 mg QE/g), followed by *E. resinifera* flowers (18.54 ± 0.06 to 40.21 ± 0.21 mg QE/g). In *E. echinus* flowers, TFC content ranged from 5.21 ± 0.16 to 32.80 ± 0.16 mg QE/g. Notably, the ethanolic extract of *E. echinus* propolis showed the lowest TFC concentration (0.32 ± 0.01), while its aqueous extract did not quantify flavonoids. The aqueous extract of *E. resinifera* propolis showed 0.14 ± 0.00 mg QE/g TFC content, with no significant difference observed in its aqueous extract of flowers and stems of *E. echinus* (Figures 2E, F and Supplementary Table S2). Honey analysis revealed substantial variations in TPC, TFC, and total condensed tannin (TCC). *Euphorbia resinifera* honey exhibited the highest values (22.31 ± 0.07 mg GAE/100g, 14.41 ± 0.01 mg QE/100g, and 4.39 ± 0.01 mg CE/100 g), while *E. echinus* honey displayed lower values (16.52 ± 0.01 mg GAE/100g, 4.76 ± 0.00 mg QE/100g, and 1.53 ± 0.00 mg CE/100 g) (Supplementary Table S3). Furthermore, all extracts of *E. resinifera*, including flowers, stems, propolis, and honey, demonstrated higher TPC values compared to *E. echinus*. These results underscore the intricate phenolic profiles within different plant parts, extracts, and honey, emphasizing the influence of extraction methods on compound yields and the potential health benefits associated with *E. resinifera*. These findings highlight the complex phenolic profiles found in diverse plant parts, extracts, and honey, underscoring the significant impact of extraction methods on compound yields. They also shed light on the potential health benefits linked to *E. resinifera*. Furthermore, the examination of phenolic compounds, crucial secondary metabolites in plants, across multiple components of both *E. resinifera* and *E. echinus*, alongside honey, reveals compelling insights into the distribution and concentrations of these compounds.

### 3.3 Antioxidant activities

**FIGURE 3:**
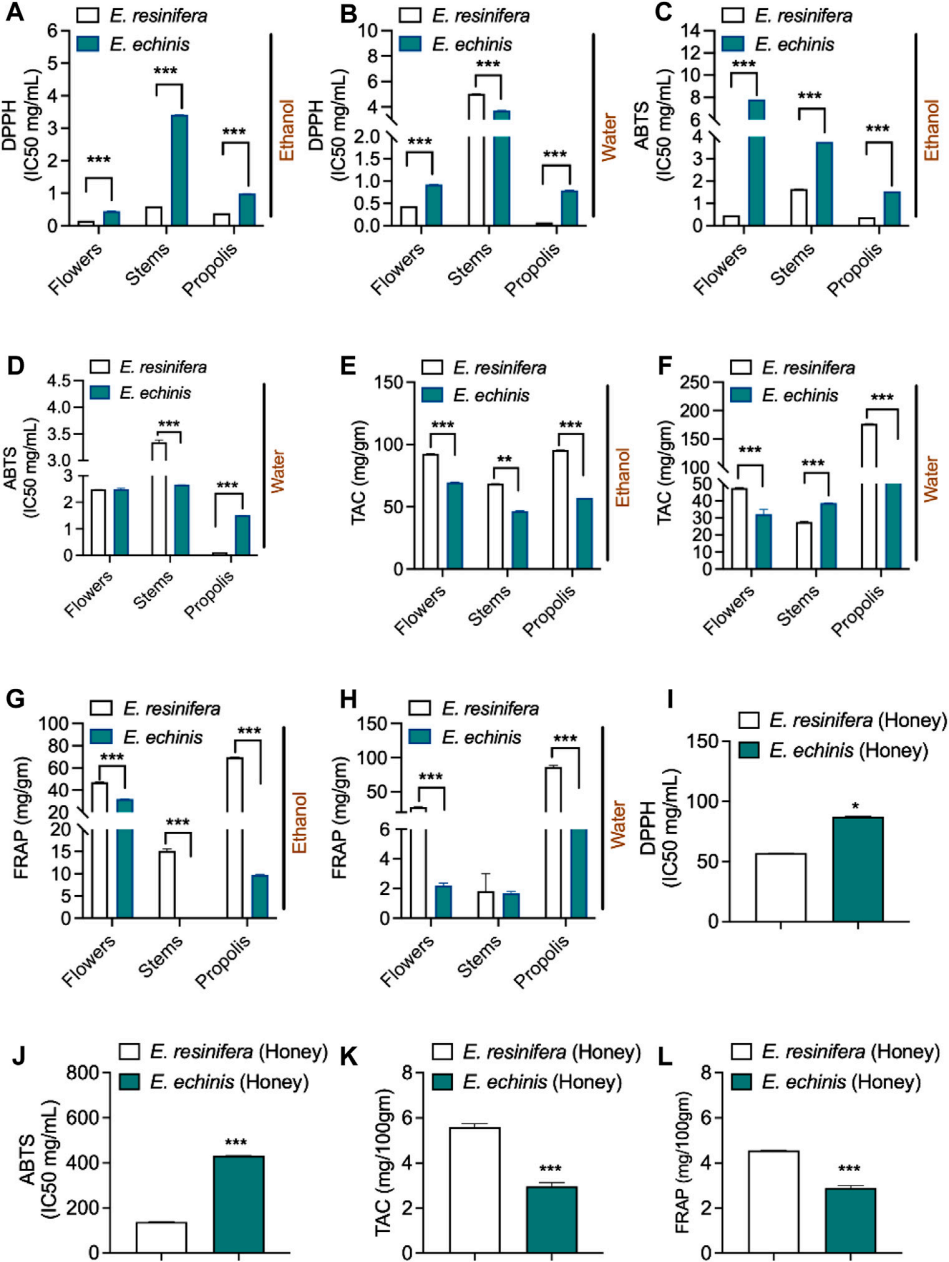
Comparison of antioxidant activity of two *Euphorbia* species. **(A)** DPPH assay of ethanolic *Euphorbia* extracts of. **(B)** DPPH assay of aqueous *Euphorbia* extract. **(C)** ABTS assay of ethanolic *Euphorbia* extracts. **(D)** ABTS assay of aqueous *Euphorbia* extracts. **(E)** TAC assay of ethanolic *Euphorbia* extracts. **(F)** TAC assay of aqueous *Euphorbia* extracts. **(G)** FRAP assay of ethanolic *Euphorbia* extracts**. (H)** FRAP assay of aqueous *Euphorbia* extracts. **(I)** DPPH assay of *Euphorbia* honey. **(J)** ABTS assay of *Euphorbia* honey. **(K)** TAC assay of *Euphorbia* honey. **(L)** FRAP assay of *Euphorbia* honey. The extraction of stems, flower propolis, and honey of *E. resinifera* and *E. echinus*. Data was displayed as the mean ± standard error (n = 3). ***p < 0.001.

To evaluate the antioxidant potential of various *Euphorbia* extracts, we conducted four distinct antioxidant tests: DPPH, ABTS, TAC, and FRAP. Notably, *E. resinifera* extracts consistently exhibited higher antioxidant activities than *E. echinus* across all assays (Figures 3A–H and Supplementary Table S4). In particular, the aqueous *E. resinifera* propolis extract demonstrated remarkable antioxidant efficacy with IC_50_ values of 0.07 ± 0.00 mg/mL and 0.13 ± 0.00 mg/mL in the DPPH and ABTS assays, respectively. These values were comparable to those exhibited by ascorbic acid (0.025 ± 0.00 mg/mL for DPPH and 0.134 ± 0.00 mg/mL for ABTS). Additionally, the *E. resinifera* aqueous propolis extract showcased the highest antioxidant capacity, recording values of (176.72 ± 0.18 mg AA/mg extract) for TAC and (86.45 ± 1.45 mg AA/mg extract) for the FRAP test. Contrastingly, in *E. echinus*, the ethanolic extract of flowers demonstrated the highest antioxidant effects, with IC_50_ values of 0.44 ± 0.00 mg/mL, 69.55 ± 0.12 mg AA/mg, and 32.03 ± 0.12 mg AA/mg in the DPPH, TAC, and FRAP assays, respectively. Interestingly, extracts from the stems exhibited the lowest antioxidant activities in both *Euphorbia* species. Further emphasizing the diversity in antioxidant profiles, *E. resinifera* honey presented an IC_50_ value of 56.71 ± 0.07 mg/mL and 137.86 ± 0.54 mg/mL for DPPH and ABTS, respectively, surpassing the antioxidant activity of *E. echinus* honey. This disparity was statistically significant (*p* < 0.001) in the analysis of the results (Figure 3I–L and Supplementary Table S5). These findings underscore the considerable antioxidant potential within *Euphorbia* extracts, with notable variations between species and different plant parts, providing valuable insights for future investigations and provide a bridge between raw data and scientific significance. Researchers can now connect observed antioxidant capacities in specific *Euphorbia* extracts to potential applications in health and industry, fostering a deeper understanding of the antioxidant landscape within this plant genus.

### 3.4 Correlation between the phenolic content and antioxidant potential of macerated extracts

The antioxidant potential is defined by the presence of certain phenolic components present in the plant. Polyphenols, flavonoids, and tannins were negatively correlated with DPPH IC_50_ (r values varied from −0.961 to −1,000, *p* < 0.01) and ABTS IC_50_ (r values varied from −0.960 to −1,000, *p* < 0.01), while they were positively correlated with TAC (r ranged from 0.959 to 1,000, *p* < 0.01) and FRAP (r varied from 0.957 to 1,000, *p* < 0.01) for all *E. resinifera* extracts. However, the correlation analysis carried out for *E. echinus* extracts provides an inverse correlation among the antioxidant activity level (ABTS and FRAP methods for stems and ABTS method for flowers) and TPC and TFC. Whereas, for propolis, the TPC has also an inverse correlation with TAC (r = −0.995, *p* < 0.01) and TFC with DPPH, ABTS and FRAP (r = −0.999, r = 0.984 and r = −0.931, *p* < 0.01 respectively) methods (Supplementary Table S6). For honey, correlations between TPC, TFC and TCC were examined using Spearman correlation (Supplementary Table S6). For *E. resinifera* honey, the excellent correlation with TPC and DPPH and FRAP (r = −1,000, r = 1,000, *p* < 0.01 respectively) was observed. For *E. echinus* honey, the highest correlation was registered between TFC, TCC and TAC assay with (r = 1,000, *p* < 0.01).

These correlation analyses unravel the intricate associations between phenolic components and antioxidant activity, providing valuable insights into the mechanisms underlying the antioxidant potential of *Euphorbia* extracts. The findings not only contribute to our understanding of plant-based antioxidants but also lay the groundwork for targeted investigations into specific phenolic compounds with potential therapeutic applications.

### 3.5 Anti-inflammatory activity

**FIGURE 4:**
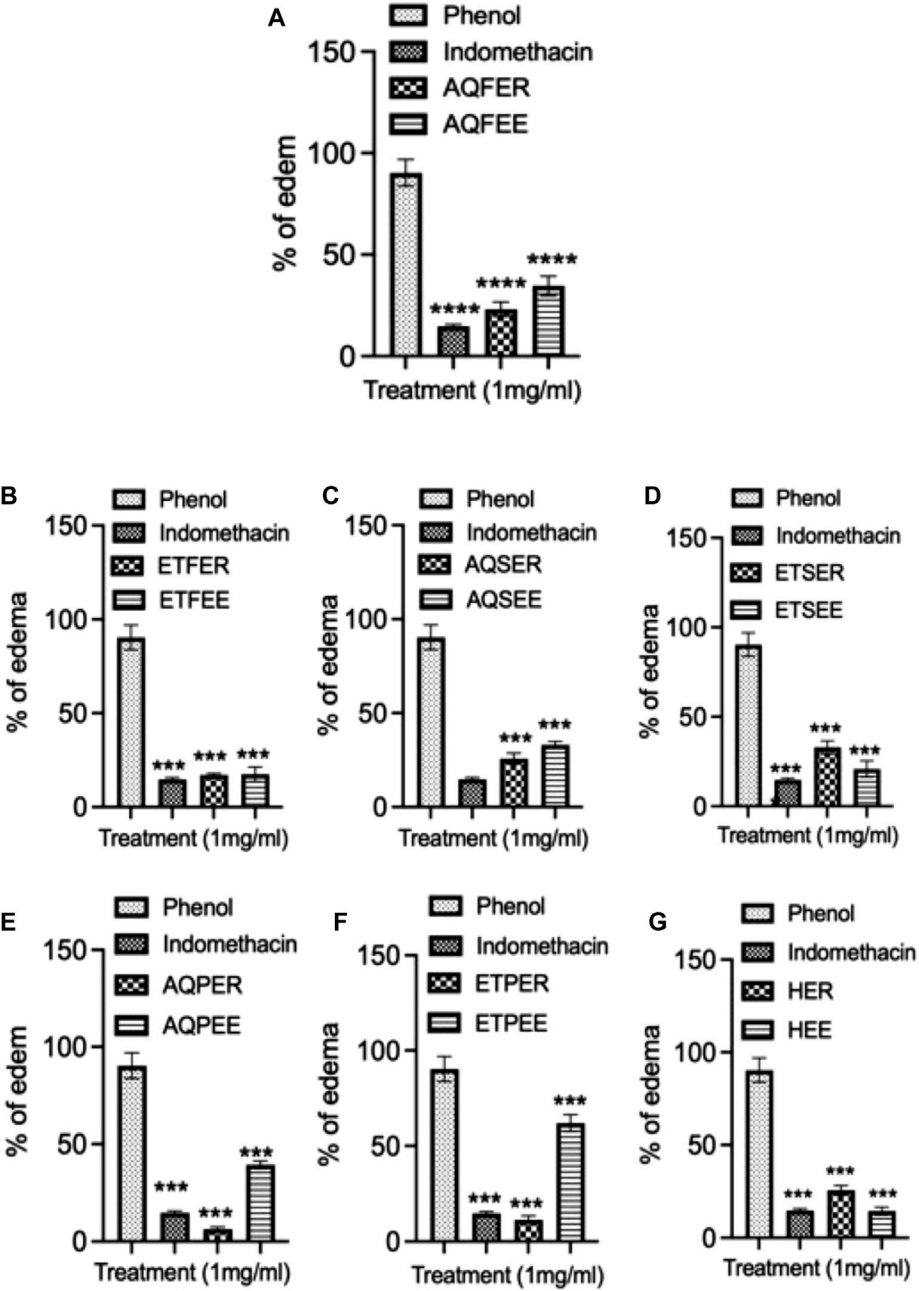
Anti-inflammatory effect by topical application of different *Euphorbia* products on phenol-induced ear edema in rats. **(A)** Anti-inflammatory effect by topical application of aqueous flower extract on phenol-induced ear edema. **(B)** Ethanol flower extract. **(C)** Aqueous stem extract. **(D)** Ethanol stem extract. **(E)** Aqueous propolis extract. **(F)** Ethanol propolis extract and **(G)** honey. Indomethacin was served as a control. Data was displayed as means ± SEM. N = 6. ***p < 0.001 and ****p ˂0.0001.

To explore the potential anti-inflammatory properties of various *Euphorbia* extracts, we conducted an experiment inducing rat ear edema using phenol, administering each extract at a concentration of 1 mg/mL. An hour after the induction of ear edema in Wistar rats, discernible alterations in ear thickness were observed in response to the application of different *Euphorbia* extracts, contrasting with the control group (Figure 4). Indomethacin, used as a positive control, demonstrated a significant reduction in edema compared to the control group, with an edema percentage of 14.64% (*p* < 0.001) (Figure 4A). Notably, the propolis extracts from *E. resinifera* exhibited remarkable anti-inflammatory potential surpassing indomethacin. The aqueous and ethanolic extracts resulted in edema percentages of 3.92% and 11.33%, respectively (Figures 4E, F). Conversely, the ethanolic extract of flowers from both *Euphorbia* species and *E. echinus* honey exhibited an effect comparable to indomethacin, with edema percentages of 14.15%, 17.43% for *E. resinifera* flowers, *E. echinus* flowers (Figure 4B), and 14.83% for *E. echinus* honey (Figure 4G). Interestingly, the ethanolic extract of *E. resinifera* stems and the aqueous extract of *E. echinus* stems displayed nearly identical anti-inflammatory effects, each yielding a percentage of edema reduction of 32.88% (Figure 4D) and 33.07% (Figure 4C), respectively. Overall, these findings underscore the diverse anti-inflammatory potentials within *Euphorbia* extracts, providing valuable insights for further exploration into their therapeutic applications and contributing to the development of novel anti-inflammatory agents.

### 3.6 Antibacterial activity

#### 3.6.1 Antibiotic susceptibility test

**FIGURE 5:**
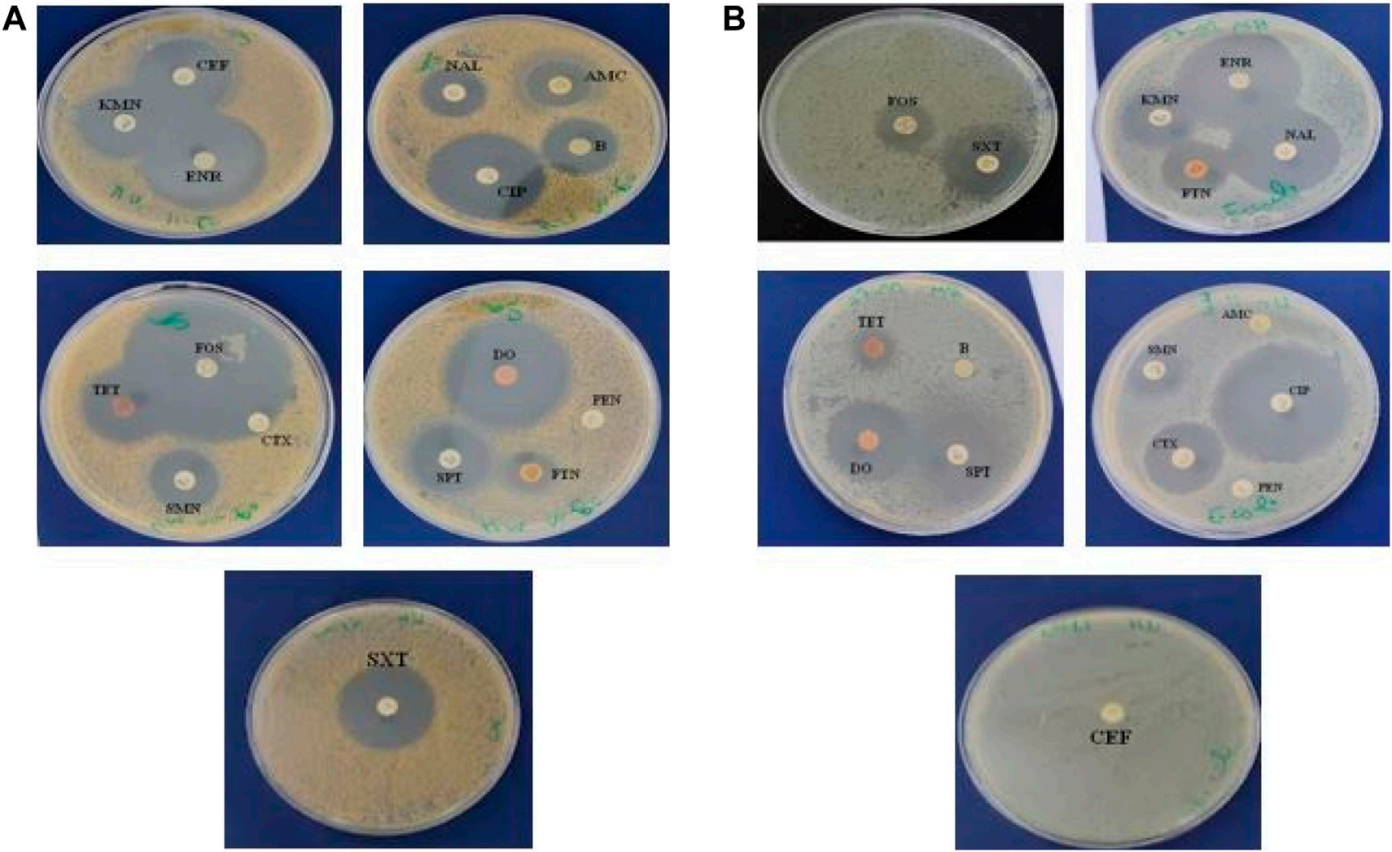
The inhibition zones (mm) of antibiotics against *S. aureus* **(A)** *and E. coli* **(B)**.

We first investigated the sensitivity of both bacterial strains (*S. aureus* and *E. coli*) to common antibiotics was tested and *E. coli* was found to be sensitive to antibiotics except for AMC, PEN, B, and CEF. However, *S. aureus* was only resistant to CTX and PEN (Table 2) and (Figure 5).

**TABLE 2:** Summary of antibiotics against and *E. coli* and *S. aureus*. The antibacterial potential was registered in the inhibition zone diameter (mm).

Antibiotic	*E. coli*	*S. aureus*
AMC	00.00 ± 0.00	18.00 ± 0.10
CTX	25.03 ± 0.15	00.00 ± 0.00
CIP	39.86 ± 0.35	35.96 ± 0.00
FOS	19.99 ± 0.12	50.12 ± 0.01
SXT	35.00 ± 0.25	32.10 ± 0.10
PEN	00.00 ± 0.00	00.00 ± 0.00
NAL	33.91 ± 0.14	19.03 ± 0.06
SPT	25.95 ± 0.18	24.09 ± 0.08
DO	27.00 ± 0.1	37.16 ± 0.04
KMN	25.01 ± 0.09	26.93 ± 0.06
FTN	20.04 ± 0.09	14.86 ± 0.14
ENR	40.00 ± 0.12	39.11 ± 0.09
B	00.00 ± 0.00	22.03 ± 0.13
TET	14.02 ± 0.09	25.96 ± 0.07
SMN	18.04 ± 0.10	21.14 ± 0.12
CEF	00.00 ± 0.00	29.08 ± 0.07

Results are expressed as mean ± standard deviation, n = 3.

AMC, Ampicillin (10 µg); CTX, Céfotaxim (30 µg); CIP, Ciprofloxacin (5 μg); FOS, Fosfomycin (50 µg); SXT, Co-trimoxazole (25 µg); PEN, Penicillin (6 µg); NAL, Nalidixic acid (30 µg); SPT, Spectinomycin (100 µg); DO, Doxycycline (30 µg); KMN, Kanamycin (30 µg); FTN, Nitrofurantoin (300 µg); ENR, Enrofloxacin (5 µg); B, Bacitracin (6 µg); TET; Tétracyclin (30 µg); SMN, Streptomycin (10 µg); CEF, Cefepim (30 µg); *E. coli*, *Escherichia coli*; *S. aureus*, *staphylococcus aureus*.

#### 3.6.2 Well diffusion method

**FIGURE 6:**
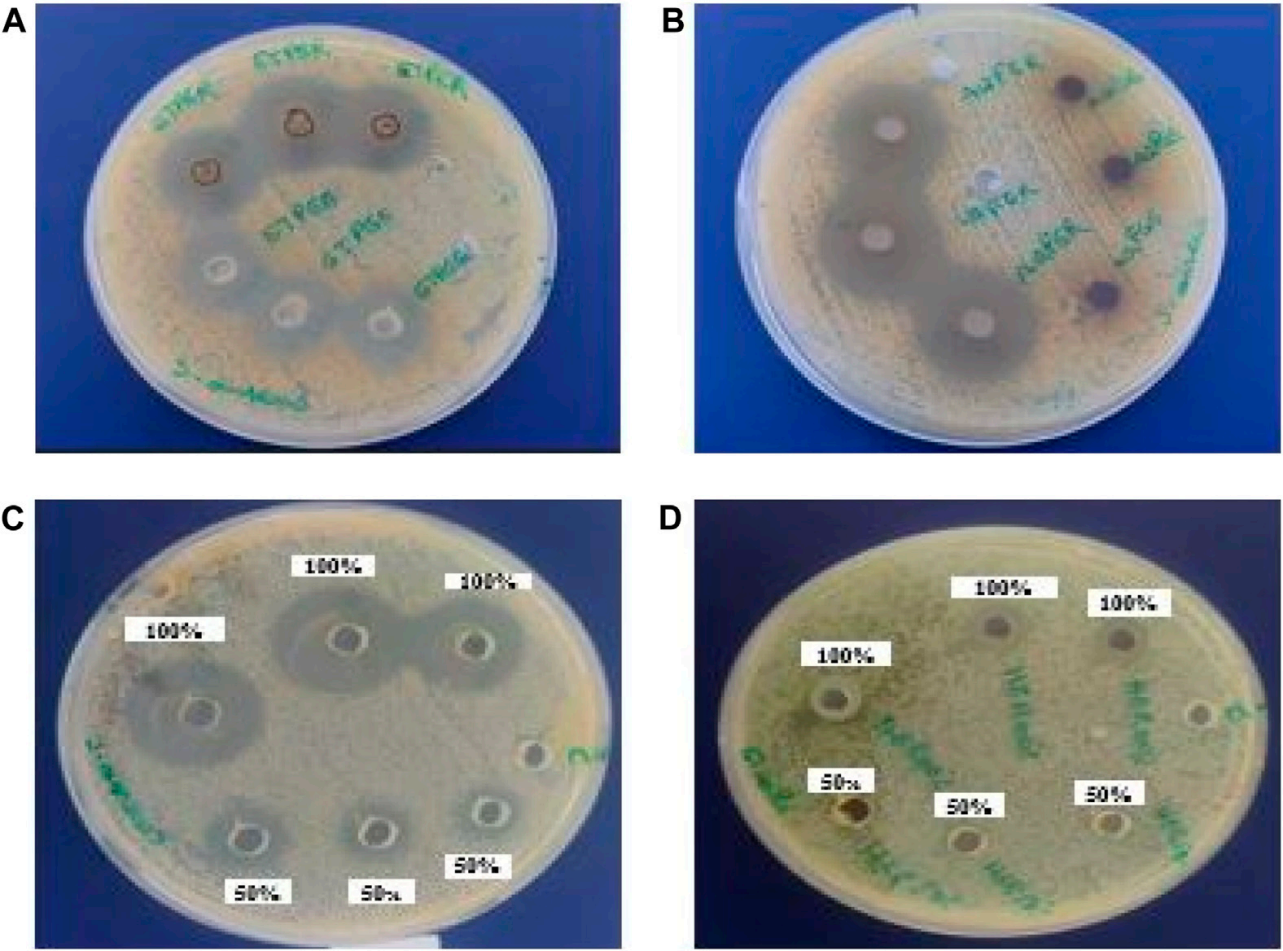
The inhibition zones (mm) of *E. resinifera* ethanol propolis extract (ETPER), *E. echinus* ethanol extract (ETPEE) **(A)**, *E. resinifera* flowers aqueous extract (AQFER) **(B)**, *E. resinifera* Honey (HER) **(C)** against *S. aureus* and *E. resinifera* honey against *E. coli* **(D)**.

The antibacterial activity of various *Euphorbia* extracts and honey was assessed in the nosocomial strains, *S. aureus* and *E. coli* by a well-diffusion method. Among all the extracts tested, only three extracts showed activity against *S. aureus.* However, no extract showed an antibacterial effect against *E. coli* (Table 3) and (Figure 6). The aqueous extract of *E. resinifera* flower showed a higher zone of inhibition (IZD) (21.0 ± 0.00 mm), followed by the ethanolic extract of *E. resinifera* propolis which with (IZD) (20.3 ± 0.00 mm) and ethanolic extract of *E. echinus* propolis (15.7 ± 0.00 mm) against *S. aureus*. The ethanolic extract of *E. echinus* propolis has an equal antibacterial effect to FTN (14.86 0.14 mm) and for *S. aureus* when compared to the antibiotics examined. The aqueous flower extract and ethanolic propolis extract of *E. resinifera* showed a higher antimicrobial potential in comparison to AMC (18.00 ± 0.10 mm), NAL (19.03 ± 0.06 mm), and FTN (14.86 ± 0.14 mm). Additionally, the agar well diffusion method was used to examine the antibacterial effects of honey from the two species of *Euphorbia* against *E. coli* and *S. aureus* at two different concentrations (50% and 100% (v/v)) (Table 4). The *E. resinifera* honey demonstrated a definite inhibition against *S. aureus* at doses of 100% and 50%, with inhibition zones of 20.7 0.03 and 11.3 0.03, respectively. Additionally, at 100% dosage, it inhibits *E. coli* growth with and 09.30 mm 0.03 mm inhibitory zone. *E. echinus* honey, however, had no impact on either of the tested strains. In essence, these results unravel the nuanced and selective antibacterial actions of *Euphorbia* extracts and honey, hinting at their potential role in targeted antibacterial therapies and contributing to the ongoing exploration of natural alternatives in combating bacterial infections.

**TABLE 3:** Antibacterial activity of the extraction of stems, flowers, and propolis of *E. resinifera* and *E. echinus* against *E. coli* and *S. aureus*. The antibacterial propeties was registered in the zone of inhibition diameter (mm).

		Zone of inhibition (mm)			
Species	Samples	*S. aureus*		*E. coli*	
		EtOH	water	EtOH	water
*E.resinifera*	Flowers	0.00 ± 0.00	21.0 ± 0.00	0.00 ± 0.00	0.00 ± 0.00
	Stems	0.00 ± 0.00	0.00 ± 0.00	0.00 ± 0.00	0.00 ± 0.00
	Propolis	20.3 ± 0.03	0.00 ± 0.00	0.00 ± 0.00	0.00 ± 0.00
*E. echinus*	Flowers	0.00 ± 0.00	0.00 ± 0.00	0.00 ± 0.00	0.00 ± 0.00
	Stems	0.00 ± 0.00	0.00 ± 0.00	0.00 ± 0.00	0.00 ± 0.00
	Propolis	15.7 ± 0.03	0.00 ± 0.00	0.00 ± 0.00	0.00 ± 0.00

*E. Euphorbia resinifera O. berg; E. echinus*, *Euphorbia officinarum subsp. Echinus (Hook.f. and Coss.) vindt; E. coli*, *Escherichia coli*; *S. aureus*, *staphylococcus aureus*.

**TABLE 4:** Antibacterial potential of *E. resinifera* and *E. echinus* honey against *E. coli* and *S. aureus*. The antibacterial activity was registered in the zone of inhibition diameter (mm).

	Zone of inhibition (mm)			
	*S. aureus*		*E. coli*	
	100%	50%	100%	50%
***E. resinifera***	20.7 ± 0.03	11.3 ± 0.03	09.30 ± 0.03	0.00 ± 0.00
***E. echinus***	0.00 ± 0.00	0.00 ± 0.00	0.00 ± 0.00	0.00 ± 0.00

*E. Euphorbia resinifera O. berg; E. echinus: Euphorbia officinarum subsp. Echinus (Hook.f. and Coss.) vindt; E. coli, Escherichia coli*; *S. aureus*, *staphylococcus aureus*.

#### 3.6.3 Minimum inhibitory concentration and minimum bactericidal concentration

The samples that displayed a zone of inhibition were then examined for their CMI and MBC in relation to the tested bacterial strains (Table 5). Except for the ethanolic extract of *E. resinifera* propolis, which showed no MIC and MBC, the aqueous extract of *E. resinifera* flowers had a bactericidal action with a MIC of 3.125 mg against *S. aureus* and an MBC of 6.25 mg/mL. With MIC values of 0.0867 mg/mL and 0.0433 mg/mL, respectively, and MBC values of 1.3875 mg/mL and 2.775 mg/mL for *S. aureus* and *E. coli*, respectively, *E. resinifera* honey demonstrated bacteriostatic activity against *S. aureus* and *E. coli*. These MIC and MBC findings not only underscore the diverse antimicrobial actions within *Euphorbia* extracts but also provide quantitative benchmarks for their efficacy against specific bacterial strains. The results contribute to the broader understanding of the therapeutic potential of these natural products in combating bacterial infections.

**TABLE 5:** Minimum inhibitory concentration (MIC) and Minimum bactericidal concentration (MBC) of different products against tested pathogen bacteria.

Samples	*S. aureus*		*E. coli*	
	MIC (mg/mL)	MBC (mg/mL)	MIC (mg/mL)	MBC (mg/mL)
***E. resinifera* flower (Water extract)**	3.125	6.25	-	-
***E. resinifera* propolis (ethanol extract)**	ND	ND	-	-
***E. echinus* propolis (ethanol extract)**	6.25	12.5	-	-
***E. resinifera* honey**	0.0867	1.3875	0.0433	2.775
***E. echinus* honey**	-	-	-	-

*E. Euphorbia resinifera O. berg; E. echinus*, *Euphorbia officinarum subsp. Echinus (Hook.f. and Coss.) vindt*; ND, not determined; *E. coli*, *Escherichia coli*; *S. aureus*, *staphylococcus aureus*. MIC, minimum inhibitory concentration; MBC, minimum bactericidal concentration.

## 4 Discussion

In recent years, the plant based metabolites utilized in the treatment of different diseases and ailments has augmented due to their health benefits and the fact that synthetic drugs cause several side effects in humans. Different techniques, including maceration, continuous hot extraction (Soxhlet), microwave-assisted extraction, decoction, and ultrasonic-assisted extraction, are employed to extract secondary metabolites (Jones and Kinghorn, 2006). Maceration, being one of the simpler methods to isolate non-volatile plant compounds, is widely applied in preliminary research (Azwanida, 2015; Zhang et al., 2018). The extraction process is influenced by various factors like the chosen method, solvent properties, solute characteristics, phytochemical constituents, extraction duration, and temperature (Do et al., 2014). *Euphorbia* products, along with differences in the polarity of the two solvents utilized Our study revealed disparities in extract yields, possibly attributable to the diverse array of phytochemicals present in different.

In order to examine the existence of phenolic content, it is imperative to analyze their occurrence in various plant components extracted using diverse organic solvents, owing to their notable biological attributes (Swamy et al., 2017). Moreover, the ethanolic extracts from the flowers and stems of both *Euphorbia* species demonstrated the highest concentration of phenolic compounds. This phenomenon can be elucidated by the capacity of polyphenols to engage in interactions, both among themselves and with other macromolecules such as carbohydrates and proteins, which are more readily soluble in ethanol compared to water (Mateus et al., 2004; Khorasani Esmaeili et al., 2015). Nonetheless, Boutoub, O et al.'s investigation on the aerial parts of the same species revealed a lower polyphenolic content extracted through decoction compared to our findings. Specifically, they reported 7.20 mg GAE/g and 11.8 mg GAE/g for *E. resinifera* and *E. echinus*, respectively, after a 1-h extraction period. However, the total phenolic content in honeys (57.6 mg GAE/100 g for *E. resinifera* and 61.8 mg GAE/100 g for *E. echinus*) was higher than what we observed in our study (Boutoub et al., 2021). It is worth noting that honey contains various non-phenolic compounds like sugars (e.g., glucose, sucrose, fructose), organic acids (e.g., ascorbic acid, citric acid), and ferrous sulfate, which could elevate absorbance values in the Folin-Ciocalteu assay, potentially leading to misleading results (Lester et al., 2012).

Elevated production of free radicals within the human body can result in harmful effects on cells and may contribute to the onset of various diseases (Swamy et al., 2015). However, antioxidants refer to chemical compounds with the capacity to mitigate a range of damage caused by free radicals (Mateus et al., 2004; Yamagishi and Matsui, 2011). The evaluation of antioxidant activity is commonly conducted through the use of straightforward and swift colorimetric methods such as DPPH, ABTS, TAC, and FRAP tests (Boligon et al., 2014). The work of LAHLOU, F. A et al., which studied the antioxidant potential of the *E. echinus* extract acquired by three methods: decoction, maceration, and by soxlet using the DPPH test, the different extracts possessed a high IC_50_ (4.57, 21.9 and 5.9 mg/mL obtained by soxhlet, maceration, and decoction respectively) value compared to our extracts (LAHLOU et al., 2014). On the other hand, it was observed that geographic origin of propolis influences their antioxidant activity, in which the IC_50_ values of Moroccan propolis extracted by maceration varying from 0.021 ± 0.01 to 1.308 ± 0.018 (mg/mL) for DPPH and 0.026 ± 0.0007 to 1.529 ± 0.015 for ABTS assay, and the total antioxidant capacity values ranged from 6.51 ± 1.8 to 80.82 ± 2.16 mg EAA/g) (El Menyiy et al., 2021). Another study conducted on various *Euphorbia* honey samples from diverse regions of Morocco discovered that the free radical scavenging ability of *E. resinifera* honey, with an IC_50_ value ranging from 78.50 ± 2.00 to 80.13 ± 1.11, exceeded that of our own honey sample. However, *E. echinus* honey showed better IC_50_ values ranging from 16.30 ± 0.41 to 75.83 ± 3.63 compared to our samples. The noted distinction could potentially stem from the climatic variations in the collection locations of the samples (Elamine et al., 2018). Our data are in close agreement with O Boutoub et al., who found that all aqueous extracts had higher amounts of phenolic compounds and potent antioxidants than honey samples (Boutoub et al., 2021). The results of this study indicate that the observed antioxidant potential in the plant extracts can be attributed to the presence of specific phenolic compounds in these plants. Particularly, polyphenols, flavonoids, and tannins have been identified as significant contributors to antioxidant activity, as indicated by their strong correlations with various antioxidant assays (Supplementary Table S1). These compounds may function as antioxidants by trapping reactive oxygen species, consequently mitigating oxidative stress and safeguarding against cellular damage.

These results are in line with the findings of earlier researchers (Mohanty et al., 2014) who established a correlation between antioxidants and phenolic extracts. Consequently, extracts exhibiting a less pronounced correlation between TPC, TFC, TCC, and antioxidant potential may owe their activity to other compounds, such as terpenoids and alkaloids, which were not within the scope of this study. However, a distinct pattern emerged when scrutinizing *E. echinus* extracts. In this context, a negative correlation was identified between the level of antioxidant activity (ABTS and FRAP methods for stems, and ABTS method for flowers) and the quantities of TPC and TFC. This implies that in *E. echinus* extracts, increased concentrations of polyphenols and flavonoids correspond to reduced antioxidant activity, as determined by ABTS and FRAP assays. Additionally, for propolis, a similar inverse correlation was found, indicating that higher TPC levels are associated with reduced TAC values and higher TFC levels are linked to lower DPPH, ABTS, and FRAP values (Supplementary Table S6).

One possible explanation for this negative correlation is the presence of other antioxidant mechanisms in the extracts. Some phenolic compounds can act as reducing agents, capable of donating electrons and effectively reducing certain reactive species (Koroleva et al., 2014). This reduction potential might not be fully captured by the DPPH and ABTS assays, leading to an inverse relationship between these antioxidant compounds and the measured radical scavenging activities. Moreover, one must acknowledge the intricate nature of the phenolic compounds existing in the extracts. Different phenolic structures may interact differently with the radicals generated by DPPH and ABTS assays, resulting in diverse outcomes in the antioxidant activity measurements.

Inflammation is the body’s natural response to injury and infection, aimed at reducing or eliminating swelling and pain (Freire and Van Dyke, 2013; Bindu et al., 2020). Hence, there is a need for the development of more potent drugs targeting the molecular inhibitors of pro-inflammatory mediators in inflammatory response. This marks the first instance where we showcase the anti-inflammatory capabilities of diverse *Euphorbia* extracts. Our findings suggest that all *Euphorbia* extracts demonstrate differing levels of anti-inflammatory activity in inhibiting phenol-induced rat ear edema. The differences observed among the extracts can be linked to the distinct concentrations of secondary metabolites present in each extract, as highlighted in studies by (Chel-Guerrero et al., 2022). Numerous investigations have affirmed the crucial role of polyphenols, including flavonoids, tannins, and phenols, in imparting anti-inflammatory propertie (Komakech et al., 2019; Bezerra et al., 2021). Specifically, certain compounds such as flavonoids possess the ability to inhibit pro-inflammatory enzymes, thereby mitigating inflammatory responses. The modulation of these inflammatory cascades may offer an explanation for the anti-inflammatory effects observed in our study. Nonetheless, additional research is required to elucidate the mechanism by which various *Euphorbia* products manifest anti-inflammatory responses, as well as to isolate the specific bioactive compound(s) akin to indomethacin.

The antibacterial activity analysis showed that only flower and propolis extracts of *E. resinifera* and *E. echinus* propolis extract which showed activity only against *S. aureus*. Knowing that only honey from *E. resinifera* showed activity against both bacteria. This could be explained by varying antibacterial effects observed in honey, and extracts of different *Euphorbia* species can be credited to variations in their chemical composition, the specificity of their antibacterial activity, the extraction method employed, and the concentration used in the experiments. Gram-positive bacteria lack an outer membrane shielding the peptidoglycan layer, which makes it easier for antimicrobial agents to penetrate. In contrast, Gram-negative bacteria have a multi-layered cell wall enclosed by a sophisticated outer cell membrane (Malanovic and Lohner, 2016). Talbaoui et al. reported that dichloromethane extracts from *E. resinifera* exhibited limited antibacterial effectiveness against *Rhodococcus* sp.*,* yielding an inhibition zone of 15 mm at a concentration of 50 μg/mL (Talbaoui et al., 2020). Convergent results were obtained in previous studies by Farah H et al. in which the methanolic and ethyl acetate extracts of the flowers, stems, and roots of *E. resinifera* at 500 mg/mL concentration exhibited activity against *S. aureus* but showed no activity against *E. coli*. With an inhibition diameter of 6.67 ± 0.33, 8.33 ± 0.67 and 9 ± 0 for flowers, stems and roots, respectively for the methanolic extract and an inhibition diameter of 7 ± 0, 10.83 ± 0.17 and 12.17 ± 0.17 mm for flowers, stems and roots respectively for ethyl acetate extract (Farah et al., 2014). In an earlier study conducted by our research team, which investigated the antibacterial activity of the same bacterial strains, it was found that 37 samples of *E. resinifera* honey from the Tadla Azillal region in Morocco demonstrated activity against both tested strains (Benjamaa et al., 2020). These findings may be attributed to the specificity of bacterial strains. The antibacterial properties of plant extracts can exhibit selectivity, and various honey varieties might target particular bacterial strains. *E. echinus* might not have been effective against the particular bacterial strains tested, whereas *E. resinifera* could have been more potent against them. Also, bacterial resistance; some bacterial strains may develop resistance to the antibacterial compounds present in *E. echinus*, which could diminish its effectiveness. According to several studies, phenolic acids, tannins, and flavonoids have the power to inhibit the growth of several microorganisms, including bacteria (Schmidt et al., 2012; Martín-García et al., 2022). In addition, other reports highlight a major role of phenolic compounds as antibacterial agents (Zhang et al., 2016). Polyphenols have the capacity to engage with elements of the bacterial cell wall composition, allowing them to hinder and regulate the formation of biofilms. Furthermore, they can impede the activity of microbial enzymes, disrupt protein regulation, and deplete bacterial cell enzymes of essential substrates and metal ions (Papuc et al., 2017). Phenolic compounds are recognized for their influence on the electron transport chain during bacterial respiration. They achieve this by causing morphological changes in bacterial cells and inhibiting bacterial enzymatic activity, including respiratory enzymes. Moreover, studies have shown that licochalcones A and C, which are natural phenols, effectively inhibit the bacterial respiratory chain by preventing the oxidation of NADH in bacterial membranes and inhibiting NADH-cytochrome c reductase (Haraguchi et al., 1998). Moreover, Konishi et al. demonstrated that tannins have the capability to diminish NADH dehydrogenase activity in a range of organisms, including Paracoccus denitrificans and *Bacillus subtilis* (KONISHI et al., 1993). Additionally, the presence of the hydroxyl (OH) functional group in polyphenolic compounds might disrupt the metabolic processes of microorganisms. This can result in the inaccessibility of minerals, vitamins, and carbohydrates to these microorganisms (El-Beltagi et al., 2022). Furthermore, beekeepers are familiar with and utilize propolis as an antiseptic within beehives. Studies have indicated that bees afflicted with Varroa mites, a prevalent parasite known to inflict harm upon hives, predominantly host Gram-positive bacteria (Bendel, 2002). This discovery could provide an additional explanation as to the effectiveness of propolis on Gram-positive bacteria and thus justify its use as an antiseptic within the hive. Also it can also be due to the different constituents of the resin, which are secreted at the base by the plants in order to protect themselves in the event of attacks by pathogenic agents (Dixon, 2001).

Our findings bear significant implications for patients grappling with inflammatory, oxidative, and infectious diseases. The anti-inflammatory properties of Euphorbia extracts suggest a potential alleviation of symptoms associated with chronic inflammatory disorders. Moreover, the demonstrated antioxidant activity provides hope for patients facing oxidative stress-related conditions, offering a natural and potentially safer alternative to synthetic drugs. The observed antibacterial effects, especially against *S. aureus*, present promising avenues for addressing bacterial infections. Overall, our study contributes to the potential development of natural and synergistic therapeutic approaches, aiming to enhance patient care and broaden treatment options for a range of health conditions.

Future research could involve investigating the potential synergistic effects of combining *Euphorbia* extracts with other natural bioactive compounds or conventional medications. Since *Euphorbia* extracts have demonstrated significant antioxidant and anti-inflammatory activities, exploring their interactions with other plant extracts or pharmaceutical drugs may lead to enhanced therapeutic outcomes. This research could involve conducting *in vitro* and *in vivo* studies to assess the combined effects on various health conditions, such as chronic inflammation, oxidative stress-related disorders, or bacterial infections. Understanding the synergistic interactions could open up new avenues for the development of combination therapies that harness the benefits of multiple bioactive compounds, potentially leading to more effective and safer treatment options for various ailments.

While our study highlights the promising bioactive properties of *Euphorbia* extracts, several limitations should be acknowledged. The focus on specific *Euphorbia* species and extraction methods may limit generalizability, and inherent variability in plant samples could impact consistency. The study’s reliance on *in vitro* assays calls for cautious translation to *in vivo* conditions. Additionally, the exclusive emphasis on phenolic compounds overlooks other potentially influential bioactive compounds. Standardizing extraction methods, expanding the scope of compounds studied, and considering *in vivo* experiments could enhance the study’s robustness.

## Data Availability

The original contributions presented in the study are included in the article/Supplementary Material, further inquiries can be directed to the corresponding authors.
