# Supplementary material for: Comparative evaluation of antioxidant activity, total phenolic content, anti-inflammatory, and antibacterial potential of Euphorbia-derived functional products
Source: Front Pharmacol. 2024 Feb 22;15:1345340. doi: 10.3389/fphar.2024.1345340 (PMC10919402; doi:10.3389/fphar.2024.1345340)
Supplement: Supplementary file 1 [file Table1.docx]

**Table S1.** **Yield obtained from the extraction of stems, flowers and propolis of *E. resinifera* and *E*. *echinus***

| Species Samples | Extraction yields (%)  EtOH extract Water extract |
| --- | --- |
| *E. resinifera* Flowers | 19.06 ± 0.02 aA 19.00 ± 0.01 aA |
| Stems | 9.89 ± 0.07 aB 14.27 ± 0.13 aB |
| Propolis | 55.00 ± 0.03 aC 2.80 ± 0.19 aC |
| *E.officinarum subsp. echinus* Flowers | 12.79 ± 0.01 bA 17.90 ± 0.16 bA |
| Stems | 6.43 ± 0.04 bB 6.56 ± 0.04 bB |
| Propolis | 28.87 ± 0.03bC 14.73 ± 0.15 bC |

Results are means ± standard deviation of three replicates.

*E. Euphorbia resinifera O. Berg; E. echinus: Euphorbia officinarum subsp. echinus (Hook.f. & Coss.) Vindt****.*** Measurements using different solvents; absolute ethanol (EtOH) and Water. Different lower case letters indicate significant differences among the products of the two species (p ≤ 0.05). Upper case letters indicate significant differences among products within the same species (p ≤ 0.05)

### Table S2. Total phenolic contents (TPC), total flavonoid contents (TFC), and total condensed tannin (TCC) of the extraction of stems, flowers, and propolis of *E. resinifera* and *E. echinus*

| Species | Samples | TPC  (mg GAE/g extract) | TFC  (mg QE/g extract) | TCC  (mg CE/g extract) |
| --- | --- | --- | --- | --- |
|  |  | **EthOH Water** | **EthOH Water** | **EthOH Water** |
| *E.resinifera* | Flowers | 69.70 ± 0.95 aA 41.12 ± 0.24 aA | 40.21± 0.21 aA 18.54 ± 0.06 aA | 1.81 ± 0.11 aA 1.04 ± 0.00 aA |
|  | Stems | 20.06 ± 0.03 aB 0.72 ± 0.18 aB | 19.34 ± 0.06 aB 0.14 ± 0.00 aB | 0.46 ± 0.00 aB 0.00 ± 0.00 aB |
|  | Propolis | 92.28 ± 0.05 aC 148.12 ± 0.14 aC | 42.55 ± 0.11 aC 43.42 ± 0.06 aC | 0.00 ± 0.00 aC 7.38 ± 0.19 aC |
| *E.officinarum subsp. echinus* | Flowers | 38.77 ± 0.14 bA 25.33 ± 0.20 bA | 32.80 ± 0.16 bA 5.21 ± 0.16 bB | 1.75 ± 0.68 aA 0.27 ± 0.00 bA |
|  | Stems | 11.11 ± 0.03 bB 10.30 ± 0.14 bB | 8.83 ± 0.24 bB 5.95 ± 0.25 bB | 0.08 ± 0.00 bB 0.46 ± 0.00 bA |
|  | Propolis | 18.99 ± 0.05 bC 23.36 ± 0.05 bC | 0.32 ± 0.01 bC 0.00 ± 0.00 bA | 0.00 ± 0.00 aB 0.00± 0.00 bA |

Results are means ± standard error of three replicates.

*E. Euphorbia resinifera O. Berg; E. echinus: Euphorbia officinarum subsp. echinus (Hook.f. & Coss.) Vindt*; TPC: Total phenolic content; TFC: Total flavonoid content; TCC: condensed tannin contents.

Different lowercase letters indicate significant differences among the products of the two species (p ≤ 0.05). Upper case letters indicate significant differences among products within the same species (p ≤ 0.05)

### *.*

**Table S3**. **Total phenolic contents (TPC), total flavonoid contents (TFC), total condensed tannin (TCC) of the *E. resinifera* and *E. echinus* honey**

| Species | TPC  (mg GAE/100 g) | TFC  (mg QE/100 g) | TCC  (mg CE/100 g) |
| --- | --- | --- | --- |
| *E. resinifera* honey | 22.31 ± 0.07 | 14.4.1 ± 0.01 | 4.39 ± 0.01 |
| *E.officinarum subsp. echinus* honey | 16.52 ± 0.01 | 4.76 ± 0.00 | 1.53 ± 0.00 |
| *P* value | <0.001 | <0.001 | <0.001 |

Results are means ± standard error of three replicates. *E. Euphorbia resinifera O. Berg; E. echinus: Euphorbia officinarum subsp. echinus (Hook.f. & Coss.) Vindt****;*** TPC: Total phenolic content; TFC: Total flavonoid content; TCC: condensed tannin contents.

| Species | Samples | DPPH  IC_50_(mg / ml) | ABTS  IC_50_ (mg /ml) | TAC  (mg AA/mg extract) | FRAP  (mg AA/mg extract) |
| --- | --- | --- | --- | --- | --- |
|  |  | **EthOH Water** | **EthOH Water** | **EthOH Water** | **EthOH Water** |
| *E.resinifera* | Flowers | 0.15 ± 0.00 aA 0.43 ± 0.00 aA | 0.47± 0.00 aA 2.48 ± 0.00 aA | 92.32 ± 0.24 aA 47.47 ± 0.16 aA | 47.29 ± 0.12 aA 27.76 ±0.08 aA |
|  | Stems | 0.59 ± 0.00 aB 5.03 ± 0.00 aB | 1.64± 0. 00 aB 3.36 ± 0.06 aB | 68.42 ± 0.07 aB 27.55± 0.21 aB | 15.01 ± 0.26 a B 01.83 ± 0.67 aB |
|  | Propolis | 0.10 ± 0.00 aC 0.07 ± 0.00 aC | 0.39 ± 0.00 aC 0.13 ± 0.00 aC | 95.44 ± 0.71 aC 176.72 ± 0.18 aC | 69.68 ± 0.10 aC 86.45 ± 1.45 aC |
| *E.officinarum subsp. echinus* | Flowers | 0.44 ± 0.00 bA 0.92 ± 0.00 bA | 7.81 ± 0.00 bA 2.52 ± 0.04 aA | 69.55 ± 0.12 bA 32.04 ± 1.66 bA | 32.03 ± 0.12 bA 02.21 ± 0.10 bA |
|  | Stems | 3.41 ± 0.00 bB 3.72± 0.01 bB | 3.77 ± 0.00 bB 2.66 ± 0.00 bB | 46.55 ± 0.10 bB 38.63 ± 0.07 bB | 00.00 ± 0.00 bB 01.67 ± 0.08 bB |
|  | Propolis | 0.98 ± 0.00 bC 0.79 ± 0.00 bC | 1.54 ± 0.00 bC 1.50 ±0.00 bC | 57.21 ± 0.00 bC 55.44 ± 0.07 bC | 09.70 ± 0.09 bC 10.47 ± 0.12 bC |

**Table S4**. **Antioxidant activity of the extraction of stems, flowers and propolis of *E. resinifera* and *E. echinus***

Results are shown as the mean± standard error (n=3). Different lowercase letters indicate significant differences among the products of the two species (p ≤ 0.05). Upper case letters indicate significant differences among products within the same species (p ≤ 0.05)

*E. Euphorbia resinifera O. Berg; E. echinus: Euphorbia officinarum subsp. echinus (Hook.f. & Coss.) Vindt*; DPPH: (2,2-diphenyl-1-picrylhydrazyl), radical scavenging activity; ABTS: 2,2'-azino-bis (3-ethylbenzothiazoline-6-sulfonic acid); FRAP: Ferric reducing antioxidant power.

**Table S5. Antioxidant activity of the *E. resinifera* and *E. echinus* honey**

| Species | DPPH  IC_50_ (mg /ml) | ABTS  IC_50_ (mg /ml) | TAC FRAP  (mg CE/100 g) (mg AA/100g) |
| --- | --- | --- | --- |
| *E. resinifera* honey | 56.71 ± 0.07 | 137.86 ± 0.54 | 5.69 ± 0.05 4.56 ± 0.01 |
| *E.officinarum subsp. echinus* honey | 87.31 ± 0.09 | 431. ± 0.76 | 2.99 ± 0.01 1.73 ± 0.06 |
| *P* value | <0.001 | <0.001 | <0.001 <0.001 |

Results are shown as the mean± standard error (n=3)

*E. Euphorbia resinifera O. Berg; E. echinus: Euphorbia officinarum subsp. echinus (Hook.f. & Coss.) Vindt*; DPPH: (2,2-diphenyl-1-picrylhydrazyl), radical scavenging activity; ABTS: 2,2'-azino-bis (3-ethylbenzothiazoline-6-sulfonic acid); FRAP: Ferric reducing antioxidant power.

**Table S6.** **Correlation between the phenolic compounds and antioxidant activity of two *Euphorbia* species.**

|  | **TAC** | **DPPH** | **ABTS** | **FRAP** |
| --- | --- | --- | --- | --- |
| ***E. resinifera*** |  |  |  |  |
| Flower  **TPC**  **TFC**  **TCC**  Stems  **TPC**  **TFC**  **TCC**  Propolis  **TPC**  **TFC**  **TCC**  ***E. echinus***  Flower  **TPC**  **TFC**  **TCC**  Stems  **TPC**  **TFC**  **TCC**  Propolis  **TPC**  **TFC**  **TCC**  ***E .resenifera* honey**  TPC  TFC  TCC  ***E. echinus* honey**  TPC  TFC  TCC | 1,000**  1,000**  0,959**  1,000**  1,000**  1,000**  1,000**  0,961**  0,998**  0,995**  0,995**  0,992**  0,946**  0,965**  -0,999**  -0,995**  0,996**  -  0.500  0.000  0.500  0.500  1,000**  1,000** | -1,000**  -1,000**  -0,961**  -1,000**  -1,000**  -1,000**  -1,000**  -0,963**  -0,999**  -0,999**  -1,000**  -0,961**  -0,939**  -0,972**  0,997**  -0,999**  0,999**  -  -1,000**  -0.866  0.500  0.000  -0.866  0.500 | -1,000**  -1,000**  -0,960**  -1,000**  -1,000**  -1,000**  -1,000**  -0,961**  -0,999**  0,999**  1,000**  -0,960**  0,939**  0,972**  -1,000**  -0,980**  0,984**  -  -0.500  -0.866  0.500  0.866  0.000  0.000 | 1,000**  1,000**  0,957**  0,994**  0,994**  0,994**  0,985**  0,964**  0,982**  1,000**  1,000**  0,996**  -0,916*  -0,972**  0,995**  0,943**  -0,931**  -  1,000**  0.866  -0.500  0.000  0.866  0.866 |

**. Correlation is significant at the 0.01 level

*E. Euphorbia resinifera O. Berg; E. echinus: Euphorbia officinarum subsp. echinus (Hook.f. & Coss.) Vindt*; TPC: Total phenolic content; TFC: Total flavonoid content; TCC: condensed tannin contents. TAC: Total antioxidant capacity; DPPH: (2,2-diphenyl-1-picrylhydrazyl), radical scavenging activity; ABTS: 2,2'-azino-bis (3-ethylbenzothiazoline-6-sulfonic acid); FRAP: Ferric reducing antioxidant power.
